# Data for the homology modelling of the red pigment-concentrating hormone receptor (Dappu-RPCHR) of the crustacean *Daphnia pulex*, and docking of its cognate agonist (Dappu-RPCH)

**DOI:** 10.1016/j.dib.2017.10.045

**Published:** 2017-10-24

**Authors:** Graham E. Jackson, Elumalai Pavadai, Gerd Gäde, Zaheer Timol, Niels H. Andersen

**Affiliations:** a Department of Chemistry, University of Cape Town, Private Bag, Rondebosch, Cape Town 7701, South Africa; b Biological Sciences, University of Cape Town, Private Bag, Rondebosch, Cape Town 7701, South Africa; c Chemistry Department, University of Washington, Seattle, WA 98195, United States

**Keywords:** Daphnia pulex, Red pigment-concentrating hormone, Homology modeling, Molecular docking

## Abstract

The data presented in this article are related to the publication “Interaction of the red pigment-concentrating hormone of the crustacean Daphnia pulex, with its cognate receptor, Dappu-RPCHR: A nuclear magnetic resonance and modeling study” (Jackson et al., 2017) [1]. This article contains the data for homology modeling of the red pigment-concentrating hormone (RPCH) receptor of the water flea, *Daphnia pulex* (Dappu-RPCHR), which was constructed from its primary sequence. This is the first 3D model of a crustacean G-protein coupled receptor. Docking of the agonist, pGlu-Val-Asn-Phe-Ser-Thr-Ser-Trp amide (Dappu-RPCH), was used to find a binding pocket on the receptor and compared to the binding pocket of the adipokinetic hormone (AKH) receptor from the malaria mosquito. Data for the receptor, with and without loop refinement, together with the docked agonist, are presented.

**Specifications Table** [*Please fill in right-hand column of the table below.*]

**Table:** 

Subject area	*Chemistry, biochemistry, physiology*
More specific subject area	*Invertebrate neuroendocrinology*
Type of data	*Table, figure, text files*
How data was acquired	*Molecular modeling, using Modeler 9v7*[2], Autodock Vina [3], GROMACS version 4.5.5 [4]
Data format	*Raw, Analyzed*
Experimental factors	*Primary sequence* Genbank (EU503126.1)
	*Template selection with* GPCR-ModSim Web server
	*Modeler 9v7* used to construct 100, 3D models of the receptor with the input parameters set to generate 100 models with loop refinement.
	Quality checked with PROCHECK [5] and ERRAT [6]
	Ligand docking using *Autodock Vina*[2] with a grid space of 44 × 24 × 40.
	Molecular dynamics with GROMACS [4]
Experimental features	*Homology modeling of primary sequence of Daphnia pulex RPCH receptor and docking of agonist*
Data source location	
Data accessibility	*Data are with the article*
Related research article	Graham E. Jackson, Elumalai Pavadai, Gerd Gäde, Zaheer Timol and Niels H. Andersen, Interaction of the red pigment-concentrating hormone of the crustacean Daphnia pulex, with its cognate receptor, Dappu-RPCHR: A nuclear magnetic resonance and modeling study.
	International Journal of Biological Macromolecules, 2017, https://doi.org/10.1016/j.ijbiomac.2017.08.103

**Value of the Data**

• This is the first model of a crustacean G-protein coupled receptor
• This is the first comparison of homology modelling of a crustacean GPCR and an insect GPCR.
• This data allows others to extend the study to other agonists and crustacean GPCRs.
• This is the first study of hormone docking to a crustacean receptor. The final docked position was very similar, but not identical, to other GPCR/ligand complexes.

## 1 Data

**Fig. 1:**
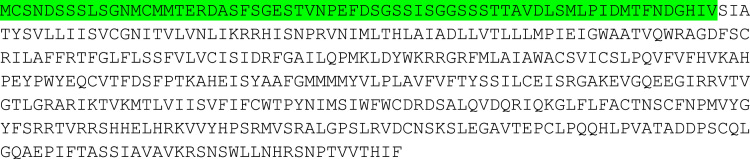
The primary sequence of Dappu-RPCHR with the signal peptide highlighted in green.

The raw data for the Dappu-RPCH receptor [1], with and without loop refinement, together with the docked agonist are given in protein database (pdb) format as supplementary data. Fig. 1 shows the primary sequence of the receptor, which was obtained from Anders [7]. This sequence compares closely to the Genbank sequence (EU503126.1) with 97.2% sequence identity. The GPCRpred server predicts that this receptor belongs to the CLASS A, rhodopsin superfamily, of G-protein coupled receptors.

**Fig. 2:**
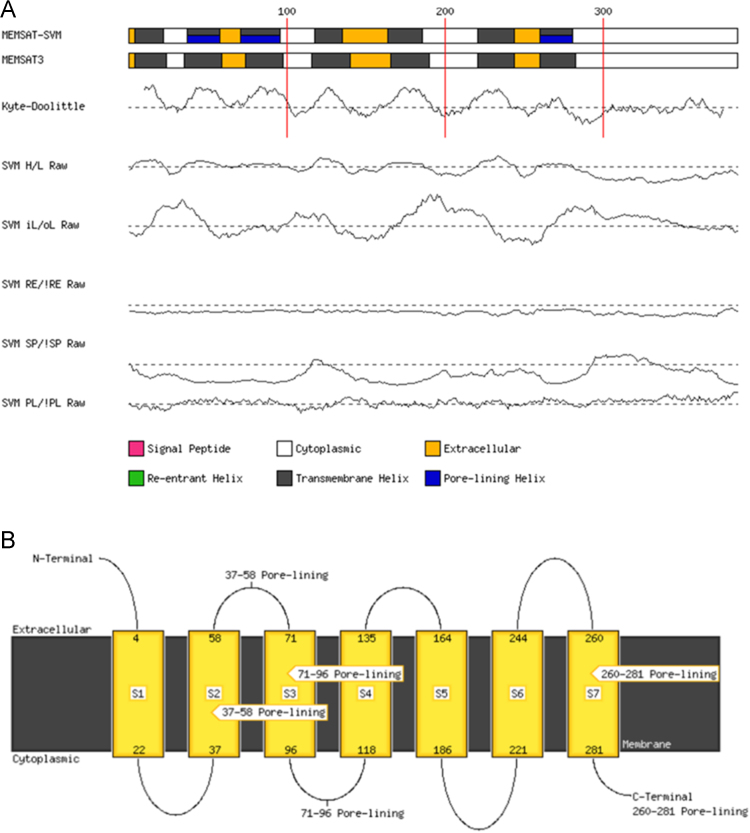
Prediction of transmembrane (TM) helices of Dappu-RPCHR by MEMSAT-SVM and MEMSAT3 servers. (a) Schematic diagram of sequence. Trace indicates the RAW output prediction threshold. PL = Pore lining residue; SP = Signal peptide residue; RE = Re-entrant helix residue; iL/oL and H/L = Helix prediction. (b) MEMSAT-SVM Cartoon.

Fig. 2 shows the MEMSAT-SVM and MEMSAT3 [8] analysis of the primary sequence data of Dappu-RPCHR. Seven transmembrane helices are predicted with a short N-terminus and long C-terminus. Helix 1 runs from residue 4–22; helix 2 residue 37–58; helix 3 residue 71 – 96; helix 4 residue 118–135; helix 5 residue 164–186; helix 6 residue 221–224 and helix 7 residue 260–281.

**Fig. 3:**
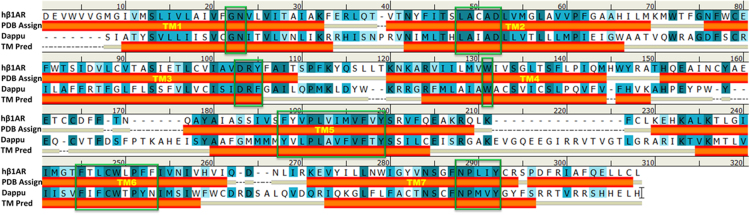
Sequence alignment of Dappu-RPCHR with the crystal structures of human β_2_-adrenergic receptor (β_2_AR).

Fig. 3 shows the sequence alignment of Dappu-RPCHR with the crystal structures of β2AR (PDB id: 2RH1). The coloring scheme indicates the degree of similarity at each alignment column; identical (strong blue background), strongly similar (light blue background), weakly similar (very light blue background) and non-matching residues (white background). Experimentally determined secondary structures for β2AR are color coded, with helices in red, strands in blue, and coils in beige. The seven transmembrane helices (TM1-TM7) are highlighted and highly conserved residues among Class A GPCRs in β2AR and Dappu-RPCHR are represented in green colored boxes.

**Fig. 4:**
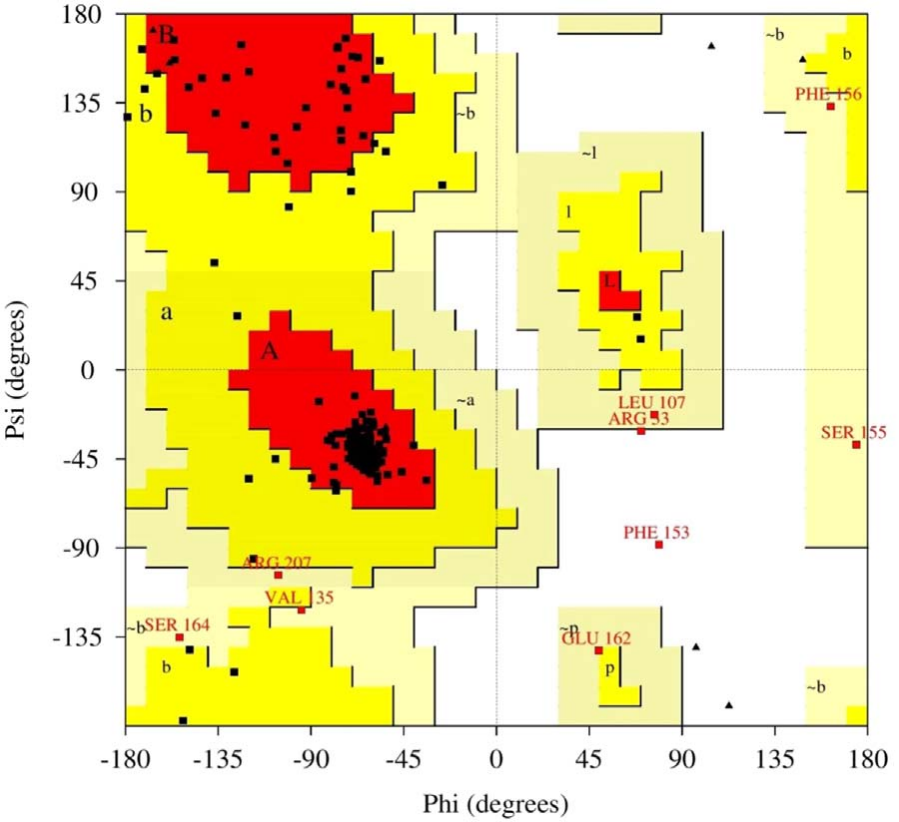
Ramachandran plot of the Dappu-RPCHR model.

Fig. 4 shows a Ramachandran plot of the Dappu-RPCHR model. Of the model residues, 89.2% occupy the core regions (red), 7.5% occupy allowed regions (yellow), 2.6% occupy generously allowed regions (light yellow), and 0.7% occupy disallowed regions (white).

**Fig. 5:**
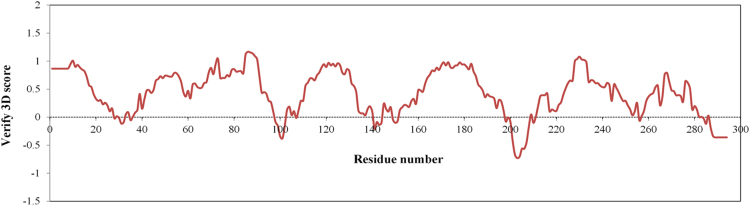
Evaluation of Dappu-RPCHR model by Verify3D program [9].

Fig. 5 shows the evaluation of the Dappu-RPCHR model by the Verify3D program [9]. Residues with positive compatibility score show that the model is reasonably folded.

**Fig. 6:**
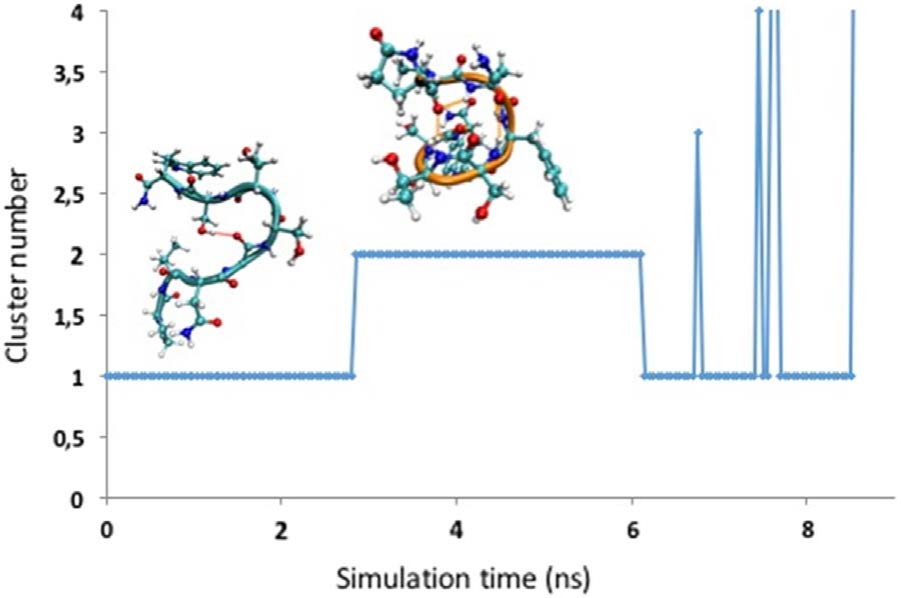
Cluster analysis of Dappu-RPCH molecular dynamics in water and 298 K.

Fig. 6 shows the molecular dynamics of the agonist, Dappu-RPCH in water. The figure shows the molecule jumping between its two major clusters.

**Fig. 7:**
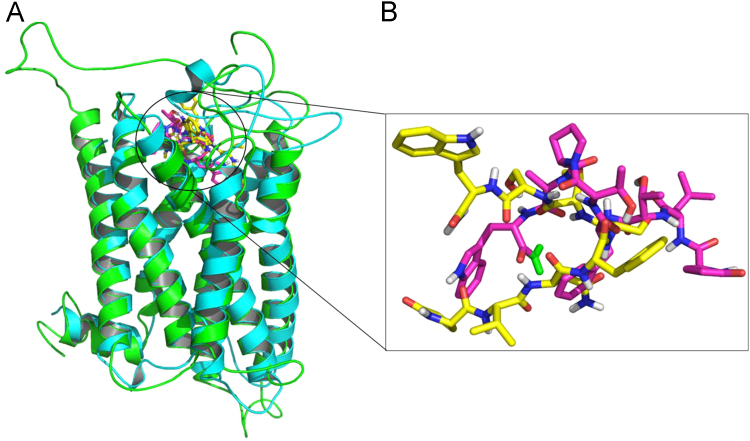
(A) Overlay of two receptors, Dappu-RPCHR (cyan) and AKHR (green), together with their hormones Dappu-RPCH (yellow) and Anoga-HrTH (magenta) [10]. (B) Overlay of Dappu-RPCH (yellow) and Anoga-HrTH (magenta).

Fig. 7 shows an overlay of the receptor Dappu-RPCHR (cyan) and the AKH receptor (green) (Anoga-HrTHR) of the malaria mosquito, A. gambiae [10]. Fig. 7(B) shows an overlay of their respective agonists, Dappu-RPCH and Anoga-HrTH.

**Fig. 8:**
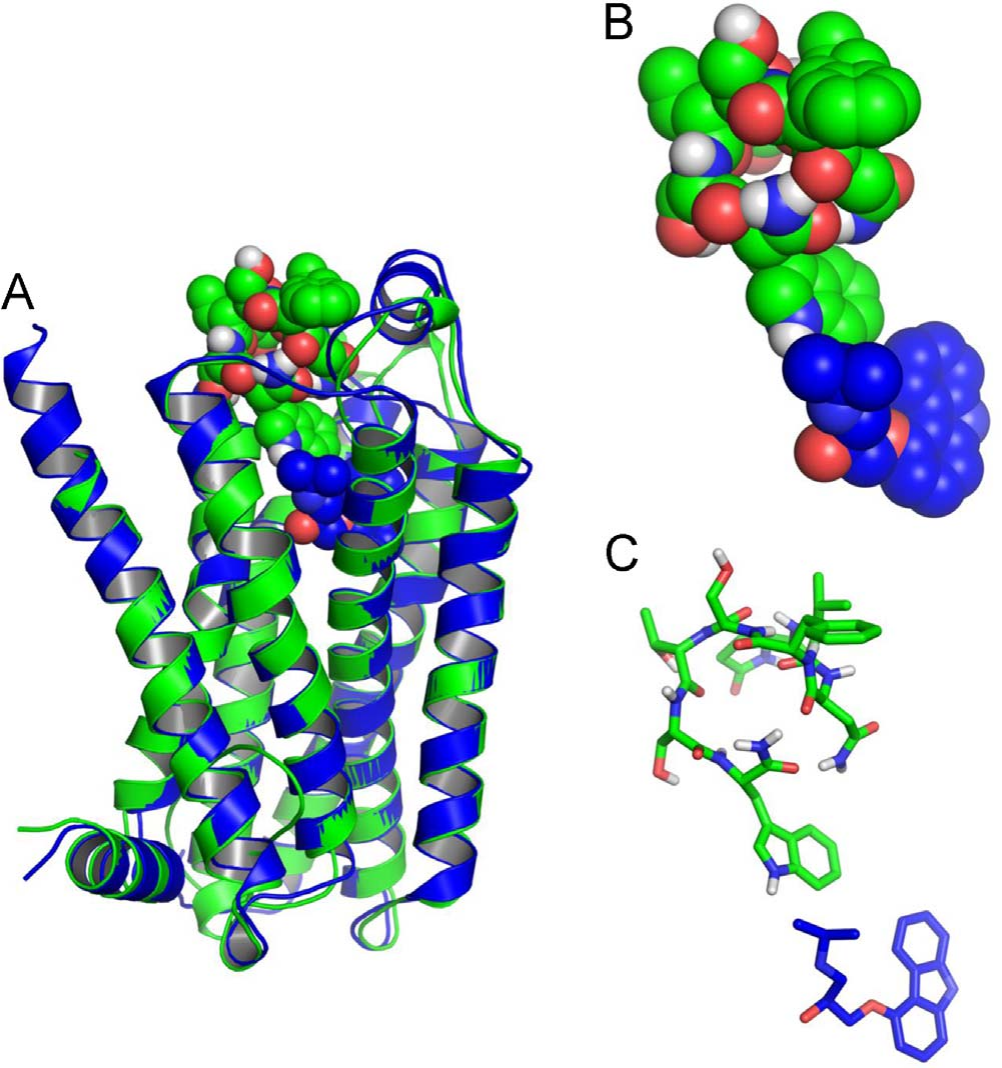
(A) Overlay of binding site of Dappu-RPCHR (green colour) and β_2_AR (blue colour) highlighting the ligands Dappu-RPCH (green) and carazolol (blue) [11]. (B) van der Waals and (C) stick representation of Dappu-RPCH (green) and carazolol (blue). Figure was prepared using PyMOL (www.pymol.com).

Since the β-adrenergic receptor (β2AR) was used as the template during the construction of Dappu-RPCHR it is interesting to compare the binding of agonists to these two receptors. Carazolol is a high affinity agonist of β2AR. Fig. 8 shows a comparison of the predicted binding sites for Dappu-RPCH in Dappu-RPCHR and carazolol in β2AR.

## 2 Experimental design, materials, and methods

The primary sequence of the Dappu-RPCH receptor was obtained from Anders [7]. The class of GPCR and trans-membrane (TM) helix predictions were computed on-line using (http://www.imtech.res.in/raghava/gpcrpred/) [12] and (http://bioinf.cs.ucl.ac.uk/psipred/) respectively.

The GPCR-ModSim Web server (http://gpcr-modsim.org/) [13] was used for template selection and preliminary sequence alignment. *Modeler 9v7* [2] was used to construct 3D models of the receptor. The quality of the constructed model was evaluated for its internal consistency and reliability using a Ramachandran plot and checking the quality of non-bonded atom interactions by ERRAT [6]. *Autodock Vina* [3] was used for peptide docking with a grid space of 44 × 24 × 40, which covered all extracellular loops and helices. The top-ranked docking poses were further optimized, using the MM-GBSA method (Prime version 2.1, Schrödinger, LLC, New York, NY, 2009).

## Appendix A Supplementary material

Dappu-RPCH docked to Dappu-RPCHR with loop refinement.

.

Dappu-RPCHR with loop refinement.

.

Dappu-RPCHR without loop refinement.
