# Supplementary material for: Data for the homology modelling of the red pigment-concentrating hormone receptor (Dappu-RPCHR) of the crustacean Daphnia pulex, and docking of its cognate agonist (Dappu-RPCH)
Source: Data Brief. 2017 Oct 24;15:941–7. doi: 10.1016/j.dib.2017.10.045 (PMC5683752; doi:10.1016/j.dib.2017.10.045)

***Conflicts of Interest Statement***

**Manuscript tit**

**le:**

**Data for the homology modelling of the red pigment-concentrating hormone receptor (Dappu-RPCHR) of the crustacean *Daphnia pulex*, and docking of its cognate agonist (Dappu-RPCH).**

The authors whose names are listed immediately below certify that they have NO affiliations with or involvement in any organization or entity with any financial interest (such as honoraria; educational grants; participation in speakers’ bureaus; membership, employment, consultancies, stock ownership, or other equity interest; and expert testimony or patent-licensing arrangements), or non-financial interest (such as personal or professional relationships, affiliations, knowledge or beliefs) in the subject matter or materials discussed in this manuscript.

## Author names:

Graham E. Jackson, Elumalai Pavadai, Gerd Gäde,

Zaheer Timol

Niels H. Andersen

**This statement is signed by the corresponding author on behalf of all the authors.**

Author's name (typed) Author's signature Date 13 October 2017

Graham E Jackson
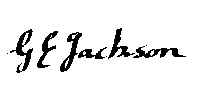

Supplement: Supplementary file 1 — Supplementary material [file mmc4.docx]
